# Osteopontin as a candidate of therapeutic application for the acute brain injury

**DOI:** 10.1111/jcmm.15641

**Published:** 2020-07-13

**Authors:** Yunxiang Zhou, Yihan Yao, Lesang Sheng, Jianmin Zhang, John H. Zhang, Anwen Shao

**Affiliations:** ^1^ Department of Surgical Oncology The Second Affiliated Hospital Zhejiang University School of Medicine Hangzhou China; ^2^ Department of Neurosurgery The Second Affiliated Hospital School of Medicine Zhejiang University Hangzhou China; ^3^ Brain Research Institute Zhejiang University Hangzhou China; ^4^ Collaborative Innovation Center for Brain Science Zhejiang University Hangzhou China; ^5^ Department of Physiology and Pharmacology Basic Sciences School of Medicine Loma Linda University Loma Linda CA USA; ^6^ Department of Anesthesiology, Neurosurgery and Neurology Loma Linda University School of Medicine Loma Linda CA USA

**Keywords:** apoptosis, intracerebral haemorrhage, neuroprotection, osteopontin, stroke, subarachnoid haemorrhage, traumatic brain injury

## Abstract

Acute brain injury is the leading cause of human death and disability worldwide, which includes intracerebral haemorrhage, subarachnoid haemorrhage, cerebral ischaemia, traumatic brain injury and hypoxia‐ischaemia brain injury. Currently, clinical treatments for neurological dysfunction of acute brain injury have not been satisfactory. Osteopontin (OPN) is a complex adhesion protein and cytokine that interacts with multiple receptors including integrins and CD44 variants, exhibiting mostly neuroprotective roles and showing therapeutic potential for acute brain injury. OPN‐induced tissue remodelling and functional repair mainly rely on its positive roles in the coordination of pro‐inflammatory and anti‐inflammatory responses, blood‐brain barrier maintenance and anti‐apoptotic actions, as well as other mechanisms such as affecting the chemotaxis and proliferation of nerve cells. The blood OPN strongly parallel with the OPN induced in the brain and can be used as a novel biomarker of the susceptibility, severity and outcome of acute brain injury. In the present review, we summarized the molecular signalling mechanisms of OPN as well as its overall role in different kinds of acute brain injury.

## 1 INTRODUCTION

Acute brain injury, exemplified by stroke, traumatic brain injury (TBI) and hypoxia‐ischaemia brain injury, is the leading cause of human death and disability worldwide.^1^, ^2^, ^3^, ^4^, ^5^ Stroke, which represents the primary reason for permanent disability in adults, can be divided into two types: ischaemic stroke, typically occurring in the setting of atherothrombosis, and haemorrhagic stroke, mainly due to the rupture of cerebral arteries.^6^, ^7^, ^8^ The latter further consists of subarachnoid haemorrhage (SAH) and intracerebral haemorrhage (ICH) and accounts for approximately 10%‐20% of strokes yet has higher mortality vs the former.^9^, ^10^, ^11^, ^12^, ^13^ TBI refers to sudden damage caused by mechanical force, occurring in traffic accidents, blast, wars, violence, terrorism, falls and sporting activity.^14^ TBI is currently the major source of fatality in young adults, with an annual global economic loss of approximately US$ 400 billion.^1^, ^2^, ^14^, ^15^, ^16^ Hypoxic‐ischaemic brain injury is another frequent, fatal and crippling neurologic disease, particularly perinatal hypoxia‐ischaemia remains the dominating cause of acute brain injury in the neonate.^17^, ^18^, ^19^ These acute brain injuries impose a heavy socio‐economic burden, whereas effective therapies are still scarce. Notably, acute neurologic disorders share many common features and processes within the pathophysiology.^20^ Although pathogenic mechanisms involved in acute brain injury have been studied extensively, which include cellular apoptosis, neuroinflammation, blood‐brain barrier (BBB) disruption,^21^ the prognosis of patients remains poor under current therapeutic strategies.^1^ New treatments targeting acute brain injury are urgently needed.

Osteopontin (OPN), a highly phosphorylated glycoprotein, is a complex adhesion protein and cytokine that interacts with multiple receptors including integrins and CD44 variants.^22^ OPN has been found in various tissues, including the brain, and plays an important role in cellular processes such as adhesion, motility and survival.^23^ Altered expression patterns of OPN have been observed in pathological conditions such as multiple sclerosis, atherosclerosis, myocardial infarction and cancers.^24^, ^25^ Under normal conditions, OPN expression is weak in the brain, while under pathological conditions including Alzheimer's disease, Parkinson's disease, TBI, stroke and hypoxia‐ischaemia brain injury, it is significantly increased in macrophages/microglia and astrocytes and exerts neuroprotective effects.^26^, ^27^, ^28^, ^29^, ^30^ In this review, we will highlight the molecular signalling pathways involved in neuroprotective part of OPN as well as its value as a potential therapeutic target, biomarker and predictor; we will also discuss the potential reason why exogenous OPN is not effective in some experimental models and put forth the limitations of current OPN research.

## 2 GENERAL FEATURES OF OPN

Osteopontin is a highly phosphorylated extracellular matrix glycoprotein that is rich in aspartic acid and has acidic characteristics consisting of approximately 314 amino acids with a molecular weight ranging between 44 and 75 kD.^31^, ^32^ OPN is initially found in osteoblasts and is later independently identified as secreted phosphoprotein 1 associated with neoplastic transformation and early T lymphocyte activation 1.^33^, ^34^, ^35^ The multiplicity of functions ascribed to OPN may reflect the presence of various isoforms, post‐translational modifications, proteolytic processing, and diversity of cell types that OPN can interact with.^32^, ^36^

OPN gene is located in the small integrin‐binding ligand, N‐linked glycoproteins (SIBLING) cluster on chromosome 4 (4q13) in the human genome and on mouse chromosome 5.^37^ The gene contains seven exons, six of which are translated in the full‐length isoform OPN‐a.^38^ Alternative translation and splicing result in two splice variants with deletion of exon 5 (OPN‐b) or deletion of exon 4 (OPN‐c), which correlates with cancer progression and poor prognosis.^38^, ^39^ Besides important isoforms, OPN is subject to significant post‐translational modifications, including phosphorylation, sulfation, glycosylation and transglutamination, and regulation of these modifications represents the potential to control OPN function.^32^, ^36^

When cleaved by thrombin, OPN transforms into two types, the N‐terminal fragment (trOPN‐N) and C‐terminal fragment (trOPN‐C).^40^ TrOPN‐N contains several highly conserved cell adhesive motifs including an Arg‐Gly‐Asp (RGD) sequence which binds to integrins such as αvβ1, αvβ3, αvβ5, α8β1 and α5β1, as well as a cryptic Ser‐Val‐Val‐Tyr‐Gly‐Leu‐Arg (SVVYGLR)‐containing domain that is only exposed after thrombin cleavage and binds to α4β1, α9β1 and α4β7 integrins,^22^ whereas trOPN‐C binds to CD44 variants.^41^ Moreover, mouse OPN cleaved by matrix metalloproteinase (MMP) 3/7 produces LRSKSRSFQVSDEQY, a novel α9β1 integrin‐binding motif in the C‐terminal fragment.^41^ In the human bone marrow, trOPN‐N constitutes the predominant form and acts as a chemotactic factor promoting hematopoietic progenitor cell homing as well as T cell‐derived IFN‐γ secretion through its binding to α9β1 and α4β1 integrins.^42^ In T cells, trOPN‐N upregulates IL‐17, whereas OPN‐C induces IL‐10 downregulation by selectively interacting with CD44 isoforms.^43^ In carotid specimens, inflammation severity is only associated with trOPN‐N expression, not with full‐length OPN or trOPN‐C.^44^ Thus, different terminal fragments may perform different functions.

## 3 THE ROLES OF OSTEOPONTIN IN ACUTE BRAIN INJURIES AND OTHER DISEASES

Osteopontin can be secreted by multiple tissues as well as body fluids and serves as a regulator in various biological mechanisms including osteoclast function, wound healing, cell migration, immune response, insulin resistance and cellular processes.^24^, ^45^, ^46^ Expression levels of OPN vary in different cell types; however, the mechanism underlying the release of OPN is not yet fully understood.^23^ In the central nervous system, OPN expression is weak under normal conditions and can be distinctly upregulated in response to either injuries or inflammation.^47^ Accumulating evidence has demonstrated that OPN plays a significant role in neurodegenerative diseases such as Alzheimer's disease, Parkinson's disease and multiple sclerosis,^26^, ^27^, ^48^ as well as acute brain injury including TBI, stroke, and hypoxia‐ischaemia brain injury.^49^, ^50^, ^51^, ^52^

Different acute brain injury models share many common features and processes within the pathophysiology. To date, most studies suggest a neuroprotective role of OPN in these neurological diseases.^49^, ^53^, ^54^, ^55^, ^56^, ^57^, ^58^, ^59^, ^60^, ^61^ Furthermore, numerous studies have demonstrated that intracerebroventricular injection or intranasal administration of exogenous OPN possesses neuroprotective roles and improves neurological outcome following ischaemic stroke and haemorrhagic stroke^53^, ^54^, ^55^, ^56^, ^57^, ^58^ (Table 1). As regards TBI, although studies have reported endogenous OPN positively influenced synapse reorganization and improved functional recovery,^49^, ^59^ the study by Jullienne et al^62^ showed exogenous administration of OPN treatment following TBI did not alter lesion characteristics. Moreover, inconsistent therapeutic effects were found in the experimental neonatal hypoxia‐ischaemia stroke treated with exogenous OPN.^60^, ^61^, ^63^, ^64^

**Table 1:** The main findings of OPN‐relevant therapeutic potential in acute brain injuries

Disease	Rat models	Agents and methods	Main findings	References
Ischaemic stroke	Male SD rats, 250‐300 g, MACO	R‐OPN, injected stereotaxically into the striatum, 1 h post‐MACO, 100 ng	Reduced the mean infarct volume, extended the therapeutic window at least to 12 h post‐MACO	Jin et al^58^
	Male SD rats, 250‐300 g, MACO	OPN peptide, intranasally, 100 ng	Reduced mean infarct volume, ameliorated neurological deficits, suppressed the induction of iNOS; the RGD motif in OPN peptide and endogenous αvβ3 integrin are essential for the neuroprotective effects	Jin et al^50^
	Male SD rats, 240‐280 g, MACO	Hyperbaric oxygen preconditioning	Improved neurological outcome, promoted expression of OPN, reduced the expression of IL‐1β/NFκB and augmented Akt phosphorylation	Hu et al^93^
	Male C57Bl/6 mice, 8 to 10 wk, transient MACO	R‐OPN, intracerebroventricularly, immediately before and immediately after surgery, 50 ng	Reduced infarct size, increased phosphorylation of Akt and p42/p44 MAPK	Meller et al^53^
Hypoxia‐ischaemia brain injury	7‐day‐old rat pups, unilateral ligation of the RCA followed by hypoxia	R‐OPN, intracerebroventricularly, 1 h post‐HI, 0.03 μg and 0.1 μg	Reduced apoptotic cell, cleaved caspase‐3 and infarct volume, ameliorated bodyweight loss, improved long‐term neurological impairment	Chen et al^61^
	Postnatal day 10 SD rat pups, unilateral ligation of the RCA followed by hypoxia	R‐OPN, intranasally, 1 h post‐HI, 5 μg	Attenuated BBB permeability and brain oedema	Dixon et al ^60^
	Postnatal day 9 C57BL/6 mice pups, permanent occlusion of the RCA followed by hypoxia	R‐OPN peptide, intranasally (350 or 2100 ng), intraperitoneally (10 mg/kg) or intracerebrally (100 ng), at several points in time	Did not exert neuroprotective effects	Bonestroo et al^63^
	Postnatal day 5 C57BL/6J mice pups, ligation of the LCA followed by hypoxia	Full‐length OPN protein and thrombin‐cleaved OPN, intranasally (1.2 μg, immediately before and after HI) and intracerebroventricularly (5 μg, immediately before HI)	Did not exert neuroprotective effects	Albertsson et al^64^
SAH	Male SD rats, 300‐350 g, endovascular perforation model	Vitamin D3, intranasally	Attenuated BBB disruption through endogenous upregulation of OPN and subsequent CD44 and P‐gp glycosylation signals in brain endothelial cells	Enkhjargal et al^127^
	Male SD rats, 300‐370 g, endovascular perforation model	R‐OPN, intracerebroventricularly, 1 h before surgery, 0.1 μg	Improved BBB disruption, increased MAPK phosphatase‐1, inactivated MAPK, reduced vascular endothelial growth factor‐A	Suzuki et al^125^
	Male SD rats, 280‐320 g, endovascular perforation model	R‐OPN, intranasally, minutes post‐SAH, 5 μg	Improved neuronal cell survival, brain oedema and neurological scores, increased p‐FAK and p‐Akt expressions, decreased caspase‐3 cleavage	Topkoru et al^55^
	Male SD rats, 250‐300 g, endovascular perforation model	Ephedra sinica extract, orally, immediately after the surgery, 15 mg/kg	Upregulated osteopontin signal, reduced the expressions of MMP‐9, alleviated the BBB disruption, improved neurological functions	Zuo et al^132^
	Male SD rats, 300‐370 g, endovascular perforation model	R‐OPN, intracerebroventricularly, 1 h pre‐SAH, 0.02 and 0.1 g	Ameliorated bodyweight loss, neurologic impairment, brain oedema and BBB disruption, inhibited NFκB and MMP‐9, maintained MMP‐1 and ZO‐1	Suzuki et al^56^
	Male SD rats, 300‐370 g, endovascular perforation model	R‐OPN, Intracerebroventricularly, 1 hour pre‐surgery or 5 hours post‐surgery, 0.01 μg, 0.02 μg or 0.1 μg	Prevented vasospasm, improved neurological impairments, inhibited MAPKs, caldesmon and HSP 27	Suzuki et al^148^
	Male SD rats, 300‐350 g, endovascular perforation model	R‐OPN, intranasally (1 h, 3 h, 6 h post‐SAH, 5 μg) and intracerebroventricularly (3 h post‐SAH, 0.1 μg)	Stabilized the phenotype of vascular smooth muscle, dilated cerebral arteries, improved neurological outcome	Wu et al^57^
	Male SD rats, 300‐375 g, the double injection model	R‐OPN, intracerebroventricularly, nearly 30 min after the first SAH, 0.3 μg or 0.1 μg	Improved neurological scores vasospasm, reduced cleaved caspase‐3, Bax and apoptosis, increased p‐Akt and Bcl‐2	He et al^109^
ICH	Male SD rats, 280‐320 g, a collagenase model	R‐OPN, intranasally, 1 h after ICH, 1μg, 3 μg or 9 μg	Attenuated brain inflammation and brain oedema improved neurological functions via integrin‐β1 induced inhibition of JAK2/STAT1 pathway	Gong et al^91^
	CD‐1 mice, 30‐40 g, a collagenase model	R‐OPN, intracerebroventricularly, 20 min pre‐ICH, 10 ng, 50 ng, or 100 ng	Improved neurological scores and brain water content	Wu et al^86^
	Male SD rats, 280‐320 g, injection of autologous blood into the right basal ganglia	R‐OPN, intracerebroventricularly, 1 h post‐ICH, 0.1 μg	Reduced neurological deficits, rotarod latencies and brain water content, increased p‐Akt expression and decreased p‐GSK‐3β, Bax/Bcl‐2 ratio and cleaved caspase‐3	Zhang et al ^99^
TBI	Male SD rats, 275‐375 g (9 to 12 wks old), moderate‐to‐severe controlled cortical impact	R‐OPN, intranasally, 1 h post‐TBI, 5 μg	Did not improve neurological score, lesion volumes, BBB dysfunction, or vascular characteristics; increased the microglial and HO‐1 response	Jullienne et al^62^
Healthy rats	Male SD rats, 270‐320 g	R‐TNC, r‐OPN, or both were injected into a cisterna magna	R‐OPN had no effect on the vessel diameter but could reverse prolonged contractions of rat basilar arteries induced by r‐TNC	Suzuki et al^146^

Abbreviations: BBB, blood‐brain barrier; FAK, focal adhesion kinase; GSK‐3β, glycogen synthase kinase 3 beta; HO‐1, haem oxygenase 1; ICH, intracerebral haemorrhage; IL, interleukin; JAK2/STAT1, Janus kinase/signal transducers and activators of transcription 1; LCA, left carotid artery; MACO, middle cerebral artery occlusion; MAPK, mitogen‐activated protein kinase; MMP, matrix metalloproteinase; NFκB, factor‐κ‐gene binding; OPN, osteopontin; RCA, right carotid artery; RGD, Arg‐Gly‐Asp; r‐OPN, recombinant; SAH, subarachnoid haemorrhage; TNC, tenascin‐C; ZO‐1, zona occludens‐1

The aforementioned experimental hypoxia‐ischaemia strokes were performed on neonatal mice or neonatal rats.^60^, ^61^, ^63^, ^64^ Accordingly, an age‐dependent manner of the neuroprotective effects of exogenous OPN has been introduced, in which OPN potentiates injury in the neonatal brain while resisting injury in the adult brain due to the different integrin subunit expression levels and distribution at different neuronal development stages ^64^ Additionally, the above models of the neuroprotective group ^60^, ^61^ and the non‐neuroprotective group ^63^, ^64^ were established on rat pups and mice pups, respectively, and consistently, the animal heterogeneity of OPN efficacy has been proposed.^62^ Jullienne et al^62^ were the first to test the function of exogenous OPN after experimental TBI on adult male SD rats (similar ages to the models in cited experimental stroke researches); although several potential beneficial alterations were observed, exogenous OPN did not substantially improve lesion characteristics. It is worthy of note that the protective effects of OPN may be overridden by the hyperacute cerebral injury but sufficient to attenuate delayed thalamic neurodegeneration.^65^

Indeed, the regulation and function of OPN may be specific to each pathophysiological condition and may be context‐dependent, spatiotemporal‐dependent or cell‐dependent, which perhaps explain the conflicting results regarding the efficacy of exogenous OPN in distinct disorders.^25^, ^27^, ^62^, ^63^, ^64^, ^65^, ^67^, ^68^, ^69^ Notably, OPN is proposed to play a dichotomous role in the neuroinflammation^70^, ^71^ (see Section 4.1 for details) and can propel the progress of various autoimmune diseases such as multiple sclerosis.^25^, ^67^ Thus, the ability to maintain the balance of anti‐inflammatory and pro‐inflammatory responses and to coordinate these signals with other inputs received by the cell (eg T cell‐independent neurodegeneration in ischaemic brain injury vs T cell‐mediated aggressive signals in multiple sclerosis contribute to the altered efficacy of OPN^65^) may define the ultimate role of OPN. Crucially, as mentioned above, different isoforms, different terminal fragments, various post‐translational modifications of OPN may yield different impact on acute brain injury.^32^, ^43^, ^44^

## 4 THE CANDIDATE MECHANISMS OF THE NEUROPROTECTIVE EFFECTS OF OSTEOPONTIN IN ACUTE BRAIN INJURY

**Figure 1:**
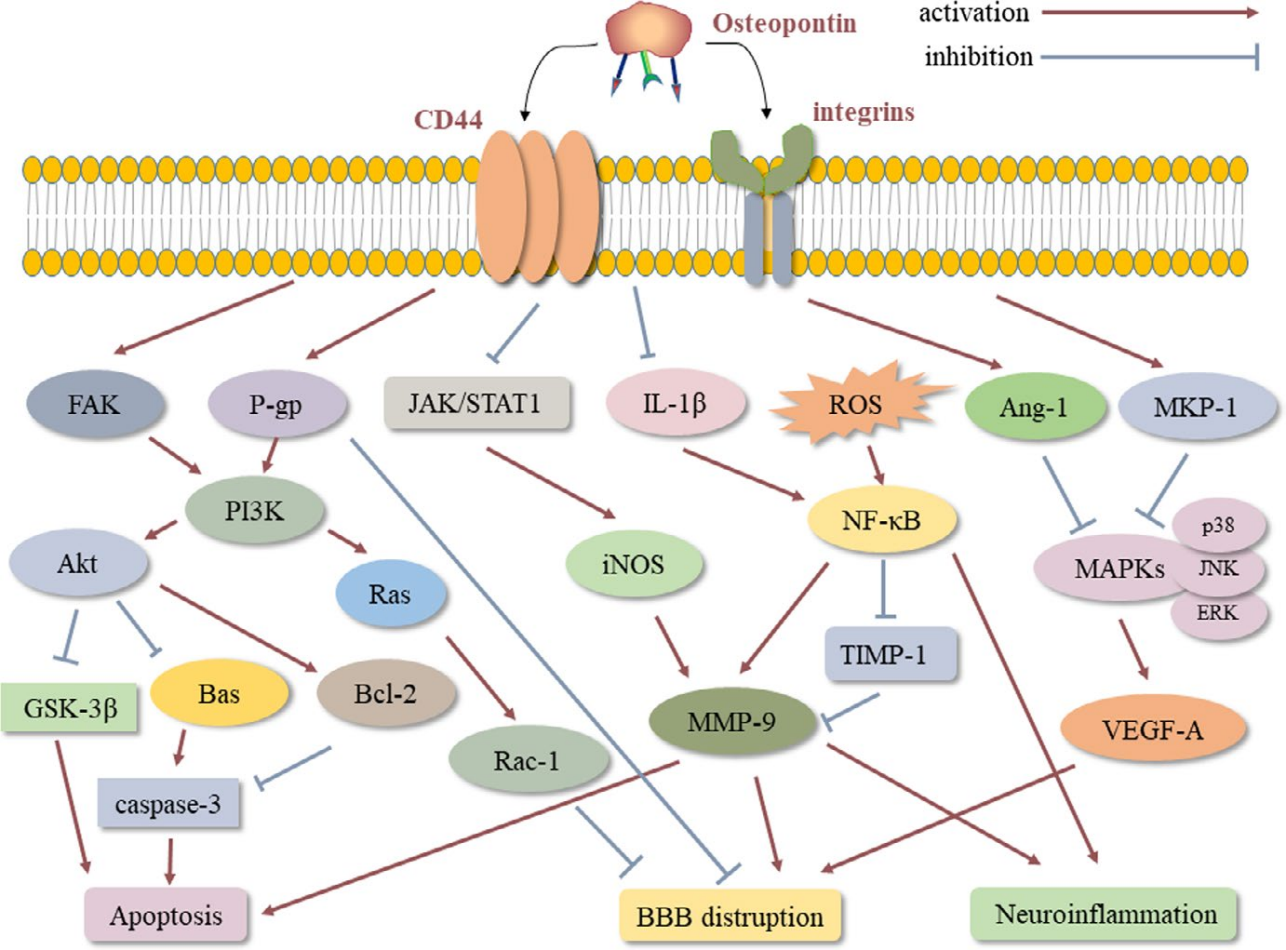
Some neuroprotective signalling pathways induced by osteopontin following acute brain injury. OPN exerts multiple roles in acute brain injury via interaction with integrins and CD44, which is reflected in its multifunctional regulation of various physiological processes, such as inflammation, apoptosis and BBB reconstruction. The mechanisms in these processes are interrelated and overlapping. AK2/STAT1, Janus kinase/signal transducers and activators of transcription 1; Ang‐1, angiopoietin‐1; BBB, blood‐brain barrier; ERK, extracellular signal‐regulated kinase; FAK, focal adhesion kinase; GSK‐3β, glycogen synthase kinase 3 beta; IL, interleukin; iNOS, inducible nitric oxide synthase; JNK, c‐Jun N‐terminal kinase; MAPK, mitogen‐activated protein kinase; MKP‐1, MAPK phosphatase‐1; MMP, matrix metalloproteinase; NFκB, factor‐κ‐gene binding; P‐gp, P‐glucoprotein; Rac‐1, Ras‐related C3 botulinum toxin substrate 1; ROS, reactive oxygen species; TIMP‐1, tissue Inhibitor of MMP‐1; VEGF, vascular endothelial growth factor

The pleiotropic effects of OPN in acute brain injury are reflected in its multifunctional regulation of various physiological processes, such as inflammation, apoptosis, BBB reconstruction and neurogenesis, and some of the mechanisms are interrelated and overlapping. In the present section, we will summarize current research findings concerning the OPN signalling in acute brain injury and integrate knowledge about its underlying mechanisms of neuroprotection and therapeutic potential. Some potential mechanisms for its beneficial effects are summarized in Figure 1, and promoting these signalling pathways may elicit a neuroprotective role of OPN.

### 4.1 OPN and neuroinflammation

Inflammation plays a crucial role in the pathogenesis of acute brain injury.^9^, ^16^, ^72^, ^73^ Potentially exacerbating secondary brain injury via inflammatory cascade in the acute stage, whereas beneficially promoting tissue remodelling and functional repair, inflammatory response induced by acute brain injury is suggested to be a double‐edged sword.^74^ By early inhibition of inflammatory cascade, the coordination of pro‐inflammatory and anti‐inflammatory responses leads to the alleviation of the brain injury and better patient outcome.^75^ Interestingly, OPN is also indicated to exert dual roles in neuroinflammation.^70^ Many studies have highlighted the pro‐inflammatory role of OPN in the pathogenesis of various autoimmune diseases, such as multiple sclerosis.^25^, ^65^, ^67^, ^76^ During acute brain injury, although some researchers have reported that OPN exacerbates cerebral injury via neuroinflammation,^71^ OPN is also considered a promising target for anti‐inflammation.^58^, ^65^, ^77^ Thus, considering the important role OPN plays in the initiation of inflammation ^70^ and its overall neuroprotective role,^49^, ^53^, ^54^, ^55^, ^56^, ^57^, ^58^, ^59^, ^60^, ^61^ OPN may maintain inflammation homeostasis with a negative feedback mechanism.

An important role of OPN in the inflammatory response is to trigger various leucocytes to cause a functional response and induce cytokine secretion, thereby forming an entire immune response.^78^ Activated microglia/macrophages and astrocytes are the main cellular sources of OPN induction in the central nervous system.^27^ Kang et al^79^ showed that microglia/macrophages and astrocytes induced OPN upregulation at different stages after brain injury. And then, the expression of OPN inversely recruited, activated and polarized additional microglial/macrophages and astrocytes in the lesional and perilesional area, which was based on the interaction of OPN with αvβ3 integrin and/or interaction with CD44.^79^, ^80^, ^81^, ^82^, ^83^ These receptors can also be upregulated after brain injury.^52^ The activated macrophages/microglia and astrocytes, as well as the induced OPN and its receptors, contribute to the succedent cytokine secretion, removal of the necrotic tissue, extracellular matrix formation, tissue remodelling, angiogenesis and gliosis.^52^, ^80^, ^84^, ^85^

Inducible nitric oxide synthase (iNOS) exerts vital roles in inflammation response and is identified to be excitotoxic and neurotoxic.^77^ Moreover, iNOS‐derived nitric oxide is known to activate MMP‐9, which is involved in neuroinflammation, cell death and the BBB disruption.^86^, ^87^, ^88^ Previous studies have demonstrated that OPN is involved in iNOS pathway.^50^, ^89^, ^90^ Induced OPN provides a dose‐dependent pattern suppressing iNOS expression after acute brain injury^65^, ^80^ by increasing expression of integrin‐β1 and then inhibiting the Janus kinase/signal transducers and activators of transcription 1 (JAK/STAT1) pathway, which is closely linked to iNOS expression.^86^, ^91^ Ladwig et al^81^ suggested that OPN downregulates iNOS by shifting the microglia phenotype towards an M2 to reduce iNOS‐expressing M1 cells. In addition to iNOS pathway, OPN also downregulates MMP‐9 expression by inhibiting interleukin (IL)‐1β/nuclear factor‐κ‐gene binding (NFκB) pathway, through which IL‐1β activates NFκB and subsequently mediates the induction of MMP‐9.^56^, ^89^, ^92^, ^93^

### 4.2 OPN and apoptosis

Apoptosis is a highly complex energy‐dependent programmed cell death process closely in correlation with neuron development and homeostasis under normal physiological conditions.^94^, ^95^ Nevertheless, apoptosis also participates in the process of neurological disorders under pathological conditions and is considered as a key player for the progression and prognosis of patients.^96^, ^97^, ^98^ Previous studies have suggested that OPN ameliorates apoptosis and promotes cell survival during brain injury through several molecular signalling pathways, which may suggest a potential therapeutic strategy for treating acute brain injury.^61^, ^99^, ^100^ And accumulating studies indicate that OPN exerts direct anti‐apoptotic actions via interaction with integrins or CD44.^53^, ^65^, ^101^, ^102^

Among all the proteins involved in the initiation and execution of apoptosis, the caspases stand out as being crucial mediators of this process.^103^, ^104^, ^105^ Furthermore, of all the caspases, caspase‐3 is probably the best understood and required for a large portion of distinct apoptotic processes and cell deaths.^104^, ^106^, ^107^ And not until caspase‐3 zymogen is cleaved by an initiator caspase following the initiation of apoptosis signal does it have activity.^106^ Thus, suppression of caspase‐3 cleavage may be effective to inhibit neuronal apoptosis.^55^, ^77^, ^108^ Consistently, experimental studies focusing on stroke and hypoxia‐ischaemia neonatal brain injury found a remarkable reduction of caspase‐3 cleavage following the OPN administration and suggested that OPN‐induced neuroprotection was associated with the inhibition of caspase‐3 activity.^61^ However, the underlying mechanisms of OPN‐induced caspase‐3 inhibition remain not entirely clear. Topkoru et al performed nasal administration of recombinant OPN 30 minutes after SAH induction on rat models, and they not only found a robust decrease of caspase‐3 cleavage but also a notable increase of phosphorylated focal adhesion kinase (FAK) and phosphorylated Akt, in line with the decline of brain oedema and improvement of neuronal cell survival and neurological status.^55^ Similarly, Zhang et al^99^ intracerebroventricularly injected recombinant OPN 1 h after ICH induced on rat models and found increased phosphorylated Akt expression and decreased phosphorylated glycogen synthase kinase 3 beta (GSK‐3β), Bax/Bcl‐2 ratio and cleaved caspase‐3, consistent with attenuated brain water content and cell death. Moreover, Meller et al and He et al also found a reduction of cleaved caspase‐3 accompanied by increased phosphorylated Akt expression and a better neurological function.^53^, ^109^

The PI3K/Akt signalling pathway is involved in numerous cellular processes, including apoptosis, BBB disruption, neurogenesis and angiogenesis.^60^, ^109^, ^110^ Inducing the phosphorylation of FAK via integrin receptor or CD44 receptor, OPN subsequently activates the PI3K/Akt signalling pathway.^60^, ^99^, ^111^ Then, phosphorylated Akt suppresses the pro‐apoptotic proteins including Bax and Bad and upregulates the anti‐apoptotic proteins such as Bcl‐2.^99^, ^109^ These molecules play important roles in the caspase‐dependent pathway, in which caspase‐3 is considered to be a common downstream protein, and the regulation of phosphorylated Akt results in the suppression of caspase‐3 and apoptosis.^61^, ^112^ In addition, phosphorylated Akt induces the phosphorylation and subsequent inactivation of GSK‐3β, which is also pro‐apoptotic and has been found to aggravate brain injury in experimental ICH, ischaemic stroke and traumatic brain injury.^113^, ^114^, ^115^ Additionally, inhibitors of FAK and PI3K administrated to the rat models could abolish the protective effects of OPN.^55^, ^99^ Thus, the PI3K/Akt pathway may play a critical role in the OPN‐induced anti‐apoptotic actions.

Besides, some proteins involved in the process of apoptosis after acute brain injury such as FAK, Bcl‐2, PI3K and Akt may overlap with autophagic pathways.^116^, ^117^ Emerging studies suggest that OPN enhances autophagy and reduces apoptosis after experimental SAH through FAK signalling, resulting in the attenuation of early brain injury and improvement of long‐term outcome.^51^

### 4.3 OPN and BBB disruption

The BBB is built up by specialized monolayer endothelial cells which are connected by tight junctions without fenestrations.^118^ The endothelial barrier is also supplemented with a large number of pericytes, which share the common basal membrane with the endothelial cells.^118^ Besides, the abluminal surface of the microvascular basement membranes is covered by astrocytic perivascular end‐feet.^119^ This major barrier plays a significant role in maintaining brain haemostasis and protecting the brain from disease and injury. Dysfunction of the BBB has been described as one of the independent risk factors for poor prognosis after acute brain injury, which may amplify inflammation and lead to further parenchyma damage and oedema by allowing more blood‐borne cells and substances to flow into the brain parenchyma.^120^, ^121^, ^122^, ^123^ Previous studies have demonstrated that OPN is significantly induced and locates at reactive capillary endothelial cells and astrocytes during the recovery of BBB function,^124^, ^125^, ^126^ suggesting an important role for OPN in BBB reconstruction. And still, OPN exerts effects of BBB maintenance via interaction with integrins and CD44 receptors.^127^

As mentioned above, MMP‐9 is involved in aggravation of BBB disruption in addition to facilitating the inflammatory cascade and cell death.^86^, ^87^, ^88^ The balanced interaction between MMP‐9 and its corresponding inhibitor, tissue inhibitor of metalloproteinase‐1 (TIMP‐1), determines the severity of BBB disruption.^128^ Induced by acute brain injury, oxidative stress and IL‐1β then activate NF‐κB, which directly upregulates MMP‐9 and inhibits TIMP‐1 levels in the brain.^56^, ^129^ There is plenty of evidence that MMP‐9 plays a role in degrading the extracellular matrix of cerebral microvessel basal lamina including laminin, fibronectin, collagen IV and zona occludens‐1 (ZO‐1) and causing BBB disruption.^130^, ^131^, ^132^ ZO‐1 belongs to endothelial tight junction‐related proteins, loss of which will increase BBB permeability.^133^ The inhibition of IL‐1β/NFκB pathway and reduction of oxidative stress have been widely reported by exogenous OPN administration, through which OPN downregulates MMP‐9 expression and upregulates TIMP‐1 to protect cerebral microvessel basal lamina and subsequently maintain BBB integrity.^56^, ^89^, ^92^, ^93^ Furthermore, the FAK/PI3K signalling pathway is also involved in BBB dysfunction following experimental brain injury.^60^ It has been demonstrated that exogenous OPN promotes the phosphorylation of FAK and subsequently activates the PI3K,^60^, ^99^, ^111^ leading to the induction of Ras‐related C3 botulinum toxin substrate 1 (Rac‐1) expression and the preservation of BBB integrity.^60^, ^134^

Interestingly, endogenous OPN induced after acute brain injury may ameliorate BBB disruption through different mechanisms. Mitogen‐activated protein kinase (MAPK) can influence the expression of vascular endothelial growth factor (VEGF)‐A, which is a potent inducer of vascular permeability.^135^, ^136^ Endogenous OPN induction after acute brain injury keeps angiopoietin (Ang)‐1 levels within the normal range, which inhibits the effect of VEGF‐A with a robust anti‐permeability property.^137^ And blockage of endogenous OPN reduces Ang‐1 and MAPK phosphatase‐1, an endogenous MAPKs inhibitor, then activates MAPKs including p38, c‐Jun N‐terminal kinase, and extracellular signal‐regulated kinase 1/2, resulting in the induction of VEGF‐A and the exacerbation of BBB permeability.^125^ In addition, endogenous OPN has been shown to induce reactive astrocyte polarization, which is pivotal to the complete neovessel coverage by astrocyte end‐feet and the integrity of BBB.^138^ Moreover, Enkhjargal et al^127^ demonstrated that brain OPN protected BBB against disruption via the CD44/P‐glucoprotein glycosylation pathway in the vascular endothelial cells.

### 4.4 Additional aspects of OPN

Previous studies also revealed that OPN exerted positive roles in the proliferation, survival and differentiation of various cells, including cerebrovascular smooth muscle cells,^138^, ^139^, ^140^ neural progenitor cells ^119^, ^141^ and neural stem cells.^142^ Moreover, adhesive and chemotactic properties of OPN exert function in the lateral migration of neuroblasts from the subventricular zone to the injured region following focal cerebral ischaemia and ICH.^143^, ^144^ OPN also reportedly enhances the sensitivity of adult corticospinal neurons to insulin‐like growth factor 1,^145^ ameliorates cerebral vasospasm ^109^, ^146^, ^147^, ^148^ and stabilizes smooth muscle cell phenotype ^57^ following acute brain injury.

## 5 OSTEOPONTIN AS A BIOMARKER OF ACUTE BRAIN INJURY

Considering the upregulation and multifarious effects of OPN following acute brain injury, it may reflect the occurrence and severity of neurological damage.^149^ And the OPN induced in the brain strongly parallels increased OPN protein in the blood, which may owe to the leakage of OPN produced in the brain or at the interface between brain and blood into the circulation.^150^, ^151^, ^152^ Thus, the plasma/serum OPN can be used as a novel biomarker of the susceptibility, severity and outcome of acute brain injury.^149^, ^153^ Nakatsuka et al^124^ demonstrated that plasma OPN levels ≥ 915.9 pmol/L at days 10‐12 was the most useful predictor of poor outcome and that plasma OPN levels ≥ 955.1 pmol/L at days 1‐3 was an independent predictor of poor outcome after SAH. Li et al^151^ suggested that plasma OPN levels at 48 h after hypoxic‐ischaemic encephalopathy were accordant with the severity of brain damage at day 7 recovery. Carbone et al^154^ showed serum OPN levels (cut‐off value, 30.53 ng/mL) were the best predictor of poor outcome at day 90 after ischaemic stroke, which is analysed by receiver operating characteristic curve, while Jing et al showed the thrombin‐cleaved OPN levels were significantly correlated with the clinical outcome 12 months after hospital discharge and could discriminate ischaemic stroke patients from healthy individuals at a cut‐off of 166.8 ng/mL.^155^ Plasma OPN levels also predict the atherosclerotic plaque destabilization.^140^, ^156^, ^157^ Ozaki et al^40^ revealed that plasma thrombin‐cleaved OPN N‐terminal levels of >5.47 pmol/L were independent predictors of atherothrombosis even within 3 h from stroke onset, thus exhibiting the early diagnostic value. The sensitivity and specificity of these cut‐off values are shown in Table 2.

**Table 2:** Cut‐off values of osteopontin as a biomarker of acute brain injuries

Study object	Osteopontin	Cut‐off values	Time‐point after stroke	Clinical value	Sensitivity (%)	Specificity (%)	References
Aneurysmal subarachnoid haemorrhage, 109 patients	Blood full‐length OPN	915.9 pmol/L	Days 10‐12	The most useful predictor of poor outcome	69.4	84.5	Nakatsuka et al^124^
		955.1 pmol/L	Days 1‐3	An independent predictor of poor outcome	65.8	70.4	
Ischaemic stroke, 90 patients	Blood full‐length OPN	30.53 ng/mL	Day 90	The best predictor of modified rankin score > 2	92	46	Carbone et al^154^
Ischaemic stroke, 377 patients; 511 healthy individuals	Blood thrombin‐cleaved OPN	166.8 ng/mL	12 mo	Discriminate ischaemic stroke patients from healthy individuals	86.3	57.7	Jing et al^155^
Ischaemic stroke, 60 patients; atherothrombotic: cardioembolic: lacunar = 28:19:13	Blood thrombin‐cleaved OPN	5.47 pmol/L	Within 24 h	Independent predictors of atherothrombosis	54	91	Ozaki et al^40^

Abbreviation: OPN, osteopontin.

## 6 CONCLUSIONS AND FUTURE PROSPECTS

In the present review, we mainly focused on the roles and therapeutic potential of OPN in acute brain injury including ICH, SAH, cerebral ischaemia, TBI and hypoxia‐ischaemia brain injury. OPN plays a bidirectional role in neuroinflammation. Following acute brain injury, OPN participates in the initiation of inflammation, meanwhile maintaining inflammation homeostasis with a negative feedback mechanism. In addition, BBB maintenance and anti‐apoptotic actions are involved in the OPN‐induced neuroprotection. Moreover, OPN exerts positive roles in the chemotaxis, proliferation, survival and differentiation of various cells, including cerebrovascular smooth muscle cells, neural progenitor cells, neural stem cells and neuroblasts. Collectively, OPN is a pleiotropic extracellular matrix glycoprotein that is mainly believed to be neuroprotective during acute brain injury. These beneficial effects of OPN are induced through its interaction with integrins and CD44. Therefore, OPN represents a potential therapeutic target for acute brain injury. Furthermore, the blood OPN strongly parallels the OPN induced in the brain and can be used as a novel biomarker of the susceptibility, severity and outcome of acute brain injury.

Notably, although endogenous OPN is believed to be neuroprotective, studies regarding TBI and neonatal hypoxia‐ischaemia brain injury suggest that exogenous OPN treatment may be ineffective or even harmful. Thus far, the reason for this discrepancy remains unclear. The efficacy of OPN may depend on the region, injury pattern and stage of brain lesions, and the age and species of victims. Additional experiments based on these variables need to be conducted to determine whether exogenous OPN can indeed activate neuroprotective signalling pathways and consequently improve neurological outcome. Furthermore, the diversity of receptors, various isoforms, post‐translational modifications and different proteolytic fragments contribute to the multiplicity of functions ascribed to OPN, which makes things more difficult to target this molecule or its putative receptors. On the contrary, precise regulation of these different types of OPN and receptors may represent the potential to control its function. Therefore, in future studies, forms of OPN and corresponding receptors should also be specified, which is not satisfactory in previous studies.

Currently, no OPN‐relevant clinical trials are being conducted. Numerous intracerebroventricular or intranasal administrations of recombinant OPN or OPN peptide in experimental models have been carried out. These administration routes, however, limit the clinical application value of OPN‐related agents, although intranasal administration of vitamin D, oral administration of Ephedra sinica extract and hyperbaric oxygen preconditioning have been shown to confer their neuroprotection through endogenous OPN signalling pathways. Besides, there is no agreement about the time window and the appropriate dose for administration hitherto. Studies concerning the protocols that are more applicable to the clinical application of OPN are warranted.

## CONFLICTS OF INTEREST

The authors declare that they have no competing interests.

## AUTHOR CONTRIBUTIONS

**Yunxiang Zhou:** Data curation (equal); Writing‐original draft (equal); Writing‐review & editing (equal). **Yihan Yao:** Writing‐review & editing (equal). **Lesang Shen:** Validation (equal); Writing‐review & editing (equal). **Jianmin Zhang:** Writing‐review & editing (equal). **John H. Zhang:** Conceptualization (equal); Writing‐review & editing (equal). **Anwen Shao:** Conceptualization (equal); Funding acquisition (equal); Writing‐review & editing (equal).

## Data Availability

No data, models or code were generated or used during the study.

## References

[1] Feigin VL , Nichols E , Alam T , et al. Global, regional, and national burden of traumatic brain injury and spinal cord injury, 1990‐2016: a systematic analysis for the Global Burden of Disease Study 2016. Lancet Neurol. 2019;18:56‐87. 30497965 10.1016/S1474-4422(18)30415-0 PMC6291456

[2] Maas AIR , Menon DK , Adelson PD , et al. Traumatic brain injury: integrated approaches to improve prevention, clinical care, and research. Lancet Neurol. 2017;16:987‐1048. 29122524 10.1016/S1474-4422(17)30371-X

[3] Wang W , Jiang B , Sun H , et al. Prevalence, incidence, and mortality of stroke in china: results from a nationwide population‐based survey of 480 687 adults. Circulation. 2017;135:759‐771. 28052979 10.1161/CIRCULATIONAHA.116.025250

[4] Thrift AG , Thayabaranathan T , Howard G , et al. Global stroke statistics. Int J Stroke. 2017;12:13‐32. 27794138 10.1177/1747493016676285

[5] Sorby‐Adams AJ , Marcoionni AM , Dempsey ER , et al. The role of neurogenic inflammation in blood‐brain barrier disruption and development of cerebral oedema following acute. Central Nervous System (CNS) Injury. Int J Mol Sci. 2017;18:1788. 10.3390/ijms18081788 PMC5578176 28817088

[6] Hademenos GJ , Massoud TF . Biophysical mechanisms of stroke. Stroke. 1997;28:2067‐2077. 9341720 10.1161/01.str.28.10.2067

[7] Rothwell PM , Coull AJ , Giles MF , et al. Change in stroke incidence, mortality, case‐fatality, severity, and risk factors in Oxfordshire, UK from 1981 to 2004 (Oxford Vascular Study). Lancet. 2004;363:1925‐1933. 15194251 10.1016/S0140-6736(04)16405-2

[8] Polivka J , Polivka J , Pesta M , et al. Risks associated with the stroke predisposition at young age: facts and hypotheses in light of individualized predictive and preventive approach. EPMA J. 2019;10:81‐99. 30984317 10.1007/s13167-019-00162-5 PMC6459458

[9] Shao A , Zhou Y , Yao Y , et al. The role and therapeutic potential of heat shock proteins in haemorrhagic stroke. J Cell Mol Med. 2019;23:5846‐5858. 31273911 10.1111/jcmm.14479 PMC6714234

[10] Sudlow CL , Warlow CP . Comparable studies of the incidence of stroke and its pathological types: results from an international collaboration. International Stroke Incidence Collaboration. Stroke. 1997;28:491‐499. 9056601 10.1161/01.str.28.3.491

[11] Gonzalez‐Perez A , Gaist D , Wallander M‐A , et al. Mortality after hemorrhagic stroke: data from general practice (The Health Improvement Network). Neurology. 2013;81:559‐565. 23843467 10.1212/WNL.0b013e31829e6eff

[12] Benjamin EJ , Blaha MJ , Chiuve SE , et al. Heart disease and stroke statistics‐2017 update: a report from the American Heart Association. Circulation. 2017;135:e146‐e603. 28122885 10.1161/CIR.0000000000000485 PMC5408160

[13] Benjamin EJ , Virani SS , Callaway CW , et al. Heart disease and stroke statistics‐2018 update: a report from the American Heart Association. Circulation. 2018;137:e67‐e492. 29386200 10.1161/CIR.0000000000000558

[14] Kardos J , Héja L , Jemnitz K , et al. The nature of early astroglial protection‐Fast activation and signaling. Prog Neurogibol. 2017;153:86‐99. 10.1016/j.pneurobio.2017.03.005 28342942

[15] An C , Jiang X , Pu H , et al. Severity‐dependent long‐term spatial learning‐memory impairment in a mouse model of traumatic brain injury. Transl Stroke Res. 2016;7:512‐520. 27539574 10.1007/s12975-016-0483-5

[16] Zhou Y , Shao A , Xu W , et al. Advance of stem cell treatment for traumatic brain injury. Front Cell Neurosci. 2019;13:301. 31456663 10.3389/fncel.2019.00301 PMC6700304

[17] Mann AP , Scodeller P , Hussain S , et al. Identification of a peptide recognizing cerebrovascular changes in mouse models of Alzheimer's disease. Nat Commun. 2017;8:1403. 29123083 10.1038/s41467-017-01096-0 PMC5680235

[18] Huang L , Zhang L . Neural stem cell therapies and hypoxic‐ischemic brain injury. Prog Neurogibol. 2019;173:1‐17. 10.1016/j.pneurobio.2018.05.004 PMC6249121 29758244

[19] Hagberg H , Mallard C , Ferriero DM , et al. The role of inflammation in perinatal brain injury. Nat Rev Neurol. 2015;11:192‐208. 25686754 10.1038/nrneurol.2015.13 PMC4664161

[20] Thelin EP , Tajsic T , Zeiler FA , et al. Monitoring the neuroinflammatory response following acute brain injury. Front Neurol. 2017;8:351. 28775710 10.3389/fneur.2017.00351 PMC5517395

[21] Yi BR , Kim SU , Choi KC . Development and application of neural stem cells for treating various human neurological diseases in animal models. Lab Anim Res. 2013;29:131‐137. 24106507 10.5625/lar.2013.29.3.131 PMC3791346

[22] Yokosaki Y , Tanaka K , Higashikawa F , et al. Distinct structural requirements for binding of the integrins alphavbeta6, alphavbeta3, alphavbeta5, alpha5beta1 and alpha9beta1 to osteopontin. Matrix Biol. 2005;24:418‐427. 16005200 10.1016/j.matbio.2005.05.005

[23] Mazzali M , Kipari T , Ophascharoensuk V , et al. Osteopontin–a molecule for all seasons. QJM. 2002;95:3‐13. 11834767 10.1093/qjmed/95.1.3

[24] Icer MA , Gezmen‐Karadag M . The multiple functions and mechanisms of osteopontin. Clin Biochem. 2018;59:17‐24. 30003880 10.1016/j.clinbiochem.2018.07.003

[25] Clemente N , Comi C , Raineri D , et al. Role of anti‐osteopontin antibodies in multiple sclerosis and experimental autoimmune encephalomyelitis. Front Immunol. 2017;8:321. 28386258 10.3389/fimmu.2017.00321 PMC5362623

[26] Wung JK , Perry G , Kowalski A , et al. Increased expression of the remodeling‐ and tumorigenic‐associated factor osteopontin in pyramidal neurons of the Alzheimer's disease brain. Curr Alzheimer Res. 2007;4:67‐72. 17316167 10.2174/156720507779939869

[27] Choi J‐S , Kim H‐Y , Cha J‐H , et al. Transient microglial and prolonged astroglial upregulation of osteopontin following transient forebrain ischemia in rats. Brain Res. 2007;1151:195‐202. 17395166 10.1016/j.brainres.2007.03.016

[28] Iczkiewicz J , Rose S , Jenner P . Osteopontin expression in activated glial cells following mechanical‐ or toxin‐induced nigral dopaminergic cell loss. Exp Neurol. 2007;207:95‐106. 17643430 10.1016/j.expneurol.2007.05.030

[29] Günther M , Plantman S , Davidsson J , et al. COX‐2 regulation and TUNEL‐positive cell death differ between genders in the secondary inflammatory response following experimental penetrating focal brain injury in rats. Acta Neurochir. 2015;157:649‐659. 25597483 10.1007/s00701-014-2331-2

[30] Gao N , Zhang‐Brotzge X , Wali B , et al. Plasma osteopontin may predict neuroinflammation and the severity of pediatric traumatic brain injury. J Cerebral Blood Flow Metab. 2020;40(1):35‐43. 10.1177/0271678X19836412 PMC6928548 30866741

[31] Clemente N , Raineri D , Cappellano G , et al. Osteopontin bridging innate and adaptive immunity in autoimmune diseases. J Immunol Res. 2016;2016:7675437. 28097158 10.1155/2016/7675437 PMC5206443

[32] Lund SA , Giachelli CM , Scatena M . The role of osteopontin in inflammatory processes. J Cell Commun Signal. 2009;3:311‐322. 19798593 10.1007/s12079-009-0068-0 PMC2778587

[33] Franzen A , Heinegard D . Isolation and characterization of two sialoproteins present only in bone calcified matrix. Biochem J. 1985;232:715‐724. 4091817 10.1042/bj2320715 PMC1152943

[34] Senger DR , Wirth DF , Hynes RO . Transformed mammalian cells secrete specific proteins and phosphoproteins. Cell. 1979;16:885‐893. 88265 10.1016/0092-8674(79)90103-x

[35] Ashkar S , Weber GF , Panoutsakopoulou V , et al. Eta‐1 (osteopontin): an early component of type‐1 (cell‐mediated) immunity. Science (New York, NY). 2000;287:860‐864. 10.1126/science.287.5454.860 10657301

[36] Christensen B , Petersen TE , Sorensen ES . Post‐translational modification and proteolytic processing of urinary osteopontin. Biochem J. 2008;411:53‐61. 18072945 10.1042/BJ20071021

[37] Sodek J , Ganss B , McKee MD . Osteopontin. Crit Rev Oral Biol Med. 2000;11:279‐303. 11021631 10.1177/10454411000110030101

[38] Gimba ER , Tilli TM . Human osteopontin splicing isoforms: known roles, potential clinical applications and activated signaling pathways. Cancer Lett. 2013;331:11‐17. 23246372 10.1016/j.canlet.2012.12.003

[39] Briones‐Orta MA , Avendaño‐Vázquez SE , Aparicio‐Bautista DI , et al. Osteopontin splice variants and polymorphisms in cancer progression and prognosis. Biochim Biophys Acta Rev Cancer. 2017;1868:93‐108.a. 28254527 10.1016/j.bbcan.2017.02.005

[40] Ozaki S , Kurata M , Kumon Y , et al. Plasma thrombin‐cleaved osteopontin as a potential biomarker of acute atherothrombotic ischemic stroke. Hypertens Res. 2017;40:61‐66. 27558929 10.1038/hr.2016.110

[41] Kon S , Nakayama Y , Matsumoto N , et al. A novel cryptic binding motif, LRSKSRSFQVSDEQY, in the C‐terminal fragment of MMP‐3/7‐cleaved osteopontin as a novel ligand for alpha9beta1 integrin is involved in the anti‐type II collagen antibody‐induced arthritis. PLoS One. 2014;9:e116210. 25545242 10.1371/journal.pone.0116210 PMC4278882

[42] Grassinger J , Haylock DN , Storan MJ , et al. Thrombin‐cleaved osteopontin regulates hemopoietic stem and progenitor cell functions through interactions with alpha9beta1 and alpha4beta1 integrins. Blood. 2009;114:49‐59. 19417209 10.1182/blood-2009-01-197988

[43] Weber GF , Ashkar S , Glimcher MJ , Cantor H . Receptor‐ligand interaction between CD44 and osteopontin (Eta‐1). Science (New York, NY). 1996;271:509‐512. 10.1126/science.271.5248.509 8560266

[44] Wolak T , Sion‐Vardi N , Novack V , et al. N‐terminal rather than full‐length osteopontin or its C‐terminal fragment is associated with carotid‐plaque inflammation in hypertensive patients. Am J Hypertens. 2013;26:326‐333. 23382482 10.1093/ajh/hps043

[45] Denhardt DT , Noda M . Osteopontin expression and function: Role in bone remodeling. J Cell Biochem. 1998;72(Suppl 30–31):92‐102. 29345811 10.1002/(SICI)1097-4644(1998)72:30/31+<92::AID-JCB13>3.0.CO;2-A

[46] Chackalaparampil I , Peri A , Nemir M , et al. Cells in vivo and in vitro from osteopetrotic mice homozygous for c‐src disruption show suppression of synthesis of osteopontin, a multifunctional extracellular matrix protein. Oncogene. 1996;12:1457‐1467. 8622862

[47] Wang KX , Denhardt DT . Osteopontin: role in immune regulation and stress responses. Cytokine Growth Factor Rev. 2008;19:333‐345. 18952487 10.1016/j.cytogfr.2008.08.001

[48] Chiocchetti A , Comi C , Indelicato M , et al. Osteopontin gene haplotypes correlate with multiple sclerosis development and progression. J Neuroimmunol. 2005;163:172‐178. 15885319 10.1016/j.jneuroim.2005.02.020

[49] Chan JL , Reeves TM , Phillips LL . Osteopontin expression in acute immune response mediates hippocampal synaptogenesis and adaptive outcome following cortical brain injury. Exp Neurol. 2014;261:757‐771. 25151457 10.1016/j.expneurol.2014.08.015 PMC4262258

[50] Jin YC , Lee H , Kim SW , et al. Intranasal delivery of RGD motif‐containing osteopontin icosamer confers neuroprotection in the postischemic brain via alphavbeta3 integrin binding. Mol Neurobiol. 2016;53:5652‐5663. 26482372 10.1007/s12035-015-9480-z

[51] Sun C , Enkhjargal B , Reis C , et al. Osteopontin‐enhanced autophagy attenuates early brain injury via FAK‐ERK pathway and improves long‐term outcome after subarachnoid hemorrhage in rats. Cells. 2019; 8:980. 10.3390/cells8090980 PMC6769958 31461955

[52] Ellison JA , Velier JJ , Spera P , et al. Osteopontin and its integrin receptor alpha(v)beta3 are upregulated during formation of the glial scar after focal stroke. Stroke. 1998;29:1698‐1706; discussion 707. 9707214 10.1161/01.str.29.8.1698

[53] Meller R , Stevens SL , Minami M , et al. Neuroprotection by osteopontin in stroke. J Cereb Blood Flow Metab. 2005;25:217‐225. 15678124 10.1038/sj.jcbfm.9600022

[54] Doyle KP , Yang T , Lessov NS , et al. Nasal administration of osteopontin peptide mimetics confers neuroprotection in stroke. J Cereb Blood Flow Metab. 2008;28:1235‐1248. 18364727 10.1038/jcbfm.2008.17 PMC6015748

[55] Topkoru BC , Altay O , Duris K , et al. Nasal administration of recombinant osteopontin attenuates early brain injury after subarachnoid hemorrhage. Stroke. 2013;44:3189‐3194. 24008574 10.1161/STROKEAHA.113.001574 PMC3919524

[56] Suzuki H , Ayer R , Sugawara T , et al. Protective effects of recombinant osteopontin on early brain injury after subarachnoid hemorrhage in rats. Crit Care Med. 2010;38:612‐618. 19851092 10.1097/CCM.0b013e3181c027ae PMC2808465

[57] Wu J , Zhang Y , Yang P , et al. Recombinant osteopontin stabilizes smooth muscle cell phenotype via integrin receptor/integrin‐linked kinase/Rac‐1 pathway after subarachnoid hemorrhage in rats. Stroke. 2016;47:1319‐1327. 27006454 10.1161/STROKEAHA.115.011552 PMC4846549

[58] Jin Y , Kim I‐Y , Kim I‐D , et al. Biodegradable gelatin microspheres enhance the neuroprotective potency of osteopontin via quick and sustained release in the post‐ischemic brain. Acta Biomater. 2014;10:3126‐3135. 24607857 10.1016/j.actbio.2014.02.045

[59] Powell MA , Black RT , Smith TL , et al. Matrix metalloproteinase 9 and osteopontin interact to support synaptogenesis in the olfactory bulb after mild traumatic brain injury. J Neurotrauma. 2019;36:1615‐1631. 30444175 10.1089/neu.2018.5994 PMC6531908

[60] Dixon B , Malaguit J , Casel D , et al. Osteopontin‐Rac1 on blood‐brain barrier stability following rodent neonatal hypoxia‐ischemia. Acta neurochirurgica Supplement. 2016;121:263‐267. 26463959 10.1007/978-3-319-18497-5_46

[61] Chen W , Ma Q , Suzuki H , et al. Osteopontin reduced hypoxia‐ischemia neonatal brain injury by suppression of apoptosis in a rat pup model. Stroke. 2011;42:764‐769. 21273567 10.1161/STROKEAHA.110.599118 PMC3045536

[62] Jullienne A , Hamer M , Haddad E , et al. Acute intranasal osteopontin treatment in male rats following TBI increases the number of activated microglia but does not alter lesion characteristics. J Neurosci Res. 2020;98(1):141–154. 30892744 10.1002/jnr.24405 PMC6754315

[63] Bonestroo HJC , Nijboer CH , van Velthoven CTJ , et al. The neonatal brain is not protected by osteopontin peptide treatment after hypoxia‐ischemia. Dev Neurosci. 2015;37:142‐152. 25765537 10.1159/000369093

[64] Albertsson A‐M , Zhang X , Leavenworth J , et al. The effect of osteopontin and osteopontin‐derived peptides on preterm brain injury. J Neuroinflammation. 2014;11:197. 25465048 10.1186/s12974-014-0197-0 PMC4266908

[65] Schroeter M , Zickler P , Denhardt DT , et al. Increased thalamic neurodegeneration following ischaemic cortical stroke in osteopontin‐deficient mice. Brain 2006;129:1426‐1437. 16636021 10.1093/brain/awl094

[66] Chiocchetti A , Indelicato M , Bensi T , et al. High levels of osteopontin associated with polymorphisms in its gene are a risk factor for development of autoimmunity/lymphoproliferation. Blood. 2004;103:1376–82. 14592838 10.1182/blood-2003-05-1748

[67] Murugaiyan G , Mittal A , Weiner HL . Increased osteopontin expression in dendritic cells amplifies IL‐17 production by CD4+ T cells in experimental autoimmune encephalomyelitis and in multiple sclerosis. J Immunol. 2008;181:7480‐7488. 19017937 10.4049/jimmunol.181.11.7480 PMC2653058

[68] Tanaka F , Ozawa Y , Inage Y , et al. Association of osteopontin with ischemic axonal death in periventricular leukomalacia. Acta Neuropathol. 2000;100:69‐74. 10912922 10.1007/s004010051194

[69] Plantman S . Osteopontin is upregulated after mechanical brain injury and stimulates neurite growth from hippocampal neurons through beta1 integrin and CD44. NeuroReport. 2012;23:647‐652. 22692550 10.1097/WNR.0b013e328355380e

[70] Shin T . Osteopontin as a two‐sided mediator in acute neuroinflammation in rat models. Acta Histochem. 2012;114:749‐754. 22947282 10.1016/j.acthis.2012.08.004

[71] Chung AG , Frye JB , Zbesko JC , et al. Liquefaction of the brain following stroke shares a similar molecular and morphological profile with atherosclerosis and mediates secondary neurodegeneration in an osteopontin‐dependent mechanism. eNeuro. 2018;5:ENEURO.0076‐18.2018. 10.1523/ENEURO.0076-18.2018 PMC6223114 30417081

[72] Jin R , Yang G , Li G . Inflammatory mechanisms in ischemic stroke: role of inflammatory cells. J Leukoc Biol. 2010;87:779‐789. 20130219 10.1189/jlb.1109766 PMC2858674

[73] Pang J , Peng J , Matei N , et al. Apolipoprotein E exerts a whole‐brain protective property by promoting M1? Microglia quiescence after experimental subarachnoid hemorrhage in mice. Transl Stroke Res. 2018;9:654‐668. 30225551 10.1007/s12975-018-0665-4

[74] Jin R , Liu L , Zhang S , et al. Role of inflammation and its mediators in acute ischemic stroke. Cardiovasc Transl Res. 2013;6:834‐851. 10.1007/s12265-013-9508-6 PMC3829610 24006091

[75] Wu L , Walas S , Leung W , et al. Neuregulin1‐beta decreases IL‐1beta‐induced neutrophil adhesion to human brain microvascular endothelial cells. Transl Stroke Res. 2015;6:116‐124. 24863743 10.1007/s12975-014-0347-9 PMC4247352

[76] Steinman L . A molecular trio in relapse and remission in multiple sclerosis. Nat Rev Immunol. 2009;9:440‐447. 19444308 10.1038/nri2548

[77] Zhu Q , Luo XU , Zhang J , et al. Osteopontin as a potential therapeutic target for ischemic stroke. Curr Drug Deliv. 2017;14:766‐772. 27848883 10.2174/1567201814666161116162148

[78] Castello LM , Raineri D , Salmi L , et al. Osteopontin at the crossroads of inflammation and tumor progression. Mediators Inflamm. 2017;2017:4049098. 28769537 10.1155/2017/4049098 PMC5523273

[79] Kang WS , Choi JS , Shin YJ , et al. Differential regulation of osteopontin receptors, CD44 and the alpha(v) and beta(3) integrin subunits, in the rat hippocampus following transient forebrain ischemia. Brain Res. 2008;1228:208‐216. 18638458 10.1016/j.brainres.2008.06.106

[80] Wang X , Louden C , Yue T‐L , et al. Delayed expression of osteopontin after focal stroke in the rat. J Neurosci. 1998;18:2075‐2083. 9482794 10.1523/JNEUROSCI.18-06-02075.1998 PMC6792923

[81] Ladwig A , Walter HL , Hucklenbroich J , et al. Osteopontin augments M2 microglia response and separates M1‐ and M2‐polarized microglial activation in permanent focal cerebral ischemia. Mediators Inflamm. 2017;2017:7189421. 29104378 10.1155/2017/7189421 PMC5632451

[82] Marcondes MCG , Poling M , Watry DD , et al. In vivo osteopontin‐induced macrophage accumulation is dependent on CD44 expression. Cell Immunol. 2008;254:56‐62. 18678363 10.1016/j.cellimm.2008.06.012 PMC2597346

[83] Lund SA , Wilson CL , Raines EW , et al. Osteopontin mediates macrophage chemotaxis via alpha4 and alpha9 integrins and survival via the alpha4 integrin. J Cell Biochem. 2013;114:1194‐1202. 23192608 10.1002/jcb.24462 PMC12462639

[84] Okada Y , Copeland BR , Hamann GF , et al. Integrin alphavbeta3 is expressed in selected microvessels after focal cerebral ischemia. Am J Pathol. 1996;149:37‐44. 8686760 PMC1865237

[85] Wan S , Cheng Y , Jin H , et al. Microglia activation and polarization after intracerebral hemorrhage in mice: the role of protease‐activated receptor‐1. Transl Stroke Res. 2016;7:478‐487. 27206851 10.1007/s12975-016-0472-8 PMC5065741

[86] Wu B , Ma Q , Suzuki H , et al. Recombinant osteopontin attenuates brain injury after intracerebral hemorrhage in mice. Neurocrit Care. 2011;14:109‐117. 20440599 10.1007/s12028-010-9372-z

[87] Chen H , Guan B , Chen XI , et al. Baicalin attenuates blood‐brain barrier disruption and hemorrhagic transformation and improves neurological outcome in ischemic stroke rats with delayed t‐PA treatment: involvement of ONOO(‐)‐MMP‐9 pathway. Transl Stroke Res. 2018;9:515‐529. 29275501 10.1007/s12975-017-0598-3

[88] Ji B , Zhou F , Han L , et al. Sodium tanshinone IIA sulfonate enhances effectiveness Rt‐PA treatment in acute ischemic stroke patients associated with ameliorating blood‐brain barrier damage. Transl Stroke Res. 2017;8:334‐340. 28243834 10.1007/s12975-017-0526-6 PMC5493726

[89] Al Dera DH . Neuroprotective effect of resveratrol against late cerebral ischemia reperfusion induced oxidative stress damage involves upregulation of osteopontin and inhibition of interleukin‐1beta. J Physiol Pharmacol. 2017;68:47‐56. 28456769

[90] Baliga SS , Merrill GF , Shinohara ML , Denhardt DT . Osteopontin expression during early cerebral ischemia‐reperfusion in rats: enhanced expression in the right cortex is suppressed by acetaminophen. PLoS One. 2011;6:e14568. 21283687 10.1371/journal.pone.0014568 PMC3024983

[91] Gong L , Manaenko A , Fan R , et al. Osteopontin attenuates inflammation via JAK2/STAT1 pathway in hyperglycemic rats after intracerebral hemorrhage. Neuropharmacology. 2018;138:160‐169. 29885817 10.1016/j.neuropharm.2018.06.009 PMC6487497

[92] Wu CY , Hsieh HL , Jou MJ , Yang CM . Involvement of p42/p44 MAPK, p38 MAPK, JNK and nuclear factor‐kappa B in interleukin‐1beta‐induced matrix metalloproteinase‐9 expression in rat brain astrocytes. J Neurochem. 2004;90:1477‐1488. 15341531 10.1111/j.1471-4159.2004.02682.x

[93] Hu S‐L , Huang Y‐X , Hu R , et al. Osteopontin mediates hyperbaric oxygen preconditioning‐induced neuroprotection against ischemic stroke. Mol Neurobiol. 2015;52:236‐243. 25146847 10.1007/s12035-014-8859-6

[94] Elmore S . Apoptosis: a review of programmed cell death. Toxicol Pathol. 2007;35:495‐516. 17562483 10.1080/01926230701320337 PMC2117903

[95] Nijhawan D , Honarpour N , Wang X . Apoptosis in neural development and disease. Annu Rev Neurosci. 2000;23:73‐87. 10845059 10.1146/annurev.neuro.23.1.73

[96] Broughton BR , Reutens DC , Sobey CG . Apoptotic mechanisms after cerebral ischemia. Stroke. 2009;40:e331‐e339. 19182083 10.1161/STROKEAHA.108.531632

[97] Gary DS , Mattson MP . Integrin signaling via the PI3‐kinase‐Akt pathway increases neuronal resistance to glutamate‐induced apoptosis. J Neurochem. 2001;76:1485‐1496. 11238733 10.1046/j.1471-4159.2001.00173.x

[98] Shi L , Al‐Baadani A , Zhou K , et al. PCMT1 Ameliorates Neuronal Apoptosis by Inhibiting the Activation of MST1 after Subarachnoid Hemorrhage in Rats. Transl Stroke Res. 2017;8:474‐483. 10.1007/s12975-017-0540-8 28534197

[99] Zhang W , Cui Y , Gao J , et al. Recombinant osteopontin improves neurological functional recovery and protects against apoptosis via PI3K/Akt/GSK‐3beta pathway following intracerebral hemorrhage. Med Sci Monit. 2018;24:1588‐1596. 29550832 10.12659/MSM.905700 PMC5870133

[100] Wu H , Niu H , Shao A , et al. Astaxanthin as a potential neuroprotective agent for neurological diseases. Marine Drugs. 2015;13:5750‐5766. 26378548 10.3390/md13095750 PMC4584352

[101] Denhardt DT , Noda M , O’Regan AW , et al. Osteopontin as a means to cope with environmental insults: regulation of inflammation, tissue remodeling, and cell survival. J Clin Investig. 2001;107:1055‐1061. 11342566 10.1172/JCI12980 PMC209291

[102] Khan SA , Lopez‐Chua CA , Zhang J , et al. Soluble osteopontin inhibits apoptosis of adherent endothelial cells deprived of growth factors. J Cell Biochem. 2002;85:728‐736. 11968013 10.1002/jcb.10170

[103] Cryns V , Yuan J . Proteases to die for. Genes Dev. 1998;12:1551‐1570. 9620844 10.1101/gad.12.11.1551

[104] Nicholson DW , Thornberry NA . Caspases: killer proteases. Trends Biochem Sci. 1997;22:299‐306. 9270303 10.1016/s0968-0004(97)01085-2

[105] Thornberry NA , Lazebnik Y . Caspases: enemies within. Science (New York, NY). 1998;281:1312‐1316. 10.1126/science.281.5381.1312 9721091

[106] Porter AG , Janicke RU . Emerging roles of caspase‐3 in apoptosis. Cell Death Differ. 1999;6:99‐104. 10200555 10.1038/sj.cdd.4400476

[107] Tulsulkar J , Glueck B , Hinds TD Jr , Shah ZA . Ginkgo biloba extract prevents female mice from ischemic brain damage and the mechanism is independent of the HO1/Wnt pathway. Transl Stroke Res. 2016;7:120‐131. 26573919 10.1007/s12975-015-0433-7 PMC4775293

[108] Manabat C , Han BH , Wendland M , et al. Reperfusion differentially induces caspase‐3 activation in ischemic core and penumbra after stroke in immature brain. Stroke. 2003;34:207‐213. 12511776 10.1161/01.STR.0000047101.87575.3C PMC2262098

[109] He J , Liu M , Liu Z , Luo L . Recombinant osteopontin attenuates experimental cerebral vasospasm following subarachnoid hemorrhage in rats through an anti‐apoptotic mechanism. Brain Res. 2015;1611:74‐83. 25779039 10.1016/j.brainres.2015.03.015

[110] Wang J , Lu Z , Fu X , et al. Alpha‐7 nicotinic receptor signaling pathway Participates in the neurogenesis induced by ChAT‐positive neurons in the subventricular zone. Transl Stroke Res. 2017;8(5):484‐493. 10.1007/s12975-017-0541-7 PMC5704989 28551702

[111] Lin YH , Yang‐Yen HF . The osteopontin‐CD44 survival signal involves activation of the phosphatidylinositol 3‐kinase/Akt signaling pathway. J Biol Chem. 2001;276:46024‐46030. 11590166 10.1074/jbc.M105132200

[112] Hasegawa Y , Suzuki H , Sozen T , et al. Apoptotic mechanisms for neuronal cells in early brain injury after subarachnoid hemorrhage. Vienna: Springer Vienna; 2011:43‐48. 10.1007/978-3-7091-0353-1_8 21116913

[113] Shapira M , Licht A , Milman A , et al. Role of glycogen synthase kinase‐3beta in early depressive behavior induced by mild traumatic brain injury. Mol Cell Neurosci. 2007;34:571‐577. 17289399 10.1016/j.mcn.2006.12.006

[114] Krafft PR , Altay O , Rolland WB , et al. alpha7 nicotinic acetylcholine receptor agonism confers neuroprotection through GSK‐3beta inhibition in a mouse model of intracerebral hemorrhage. Stroke. 2012;43:844‐850. 22207510 10.1161/STROKEAHA.111.639989 PMC3293395

[115] Valerio A , Bertolotti P , Delbarba A , et al. Glycogen synthase kinase‐3 inhibition reduces ischemic cerebral damage, restores impaired mitochondrial biogenesis and prevents ROS production. J Neurochem. 2011;116:1148‐1159. 21210815 10.1111/j.1471-4159.2011.07171.x

[116] Gabryel B , Kost A , Kasprowska D . Neuronal autophagy in cerebral ischemia–a potential target for neuroprotective strategies? Pharmacol Rep. 2012;64:1‐15. 22580515 10.1016/s1734-1140(12)70725-9

[117] Chen W , Sun Y , Liu K , Sun X . Autophagy: a double‐edged sword for neuronal survival after cerebral ischemia. Neural Regen Res. 2014;9:1210‐1216. 25206784 10.4103/1673-5374.135329 PMC4146291

[118] Keaney J , Campbell M . The dynamic blood‐brain barrier. FEBS J. 2015;282:4067‐4079. 26277326 10.1111/febs.13412

[119] Kalluri HS , Dempsey RJ . Osteopontin increases the proliferation of neural progenitor cells. Int J Develop Neurosci. 2012;30:359‐362. 10.1016/j.ijdevneu.2012.04.003 22542685

[120] Claassen J , Carhuapoma JR , Kreiter KT , et al. Global cerebral edema after subarachnoid hemorrhage: frequency, predictors, and impact on outcome. Stroke. 2002;33:1225‐1232. 11988595 10.1161/01.str.0000015624.29071.1f

[121] Pang J , Chen Y , Kuai LI , et al. Inhibition of blood‐brain barrier disruption by an apolipoprotein e‐mimetic peptide ameliorates early brain injury in experimental subarachnoid hemorrhage. Transl Stroke Res. 2017;8:257‐272. 27796945 10.1007/s12975-016-0507-1

[122] Hom S , Egleton RD , Huber JD , Davis TP . Effect of reduced flow on blood‐brain barrier transport systems. Brain Res. 2001;890:38‐48. 11164767 10.1016/s0006-8993(00)03027-4

[123] Garbuzova‐Davis S , Rodrigues MCO , Hernandez‐Ontiveros DG , et al. Blood‐brain barrier alterations provide evidence of subacute diaschisis in an ischemic stroke rat model. PLoS One. 2013;8:e63553. 23675488 10.1371/journal.pone.0063553 PMC3651135

[124] Nakatsuka Y , Shiba M , Nishikawa H , et al. Acute‐phase plasma osteopontin as an independent predictor for poor outcome after aneurysmal subarachnoid hemorrhage. Mol Neurobiol. 2018;55:6841‐6849. 29353454 10.1007/s12035-018-0893-3

[125] Suzuki H , Hasegawa Y , Kanamaru K , Zhang JH . Mechanisms of osteopontin‐induced stabilization of blood‐brain barrier disruption after subarachnoid hemorrhage in rats. Stroke. 2010;41:1783‐1790. 20616319 10.1161/STROKEAHA.110.586537 PMC2923856

[126] Iwanaga Y , Ueno M , Ueki M , et al. The expression of osteopontin is increased in vessels with blood‐brain barrier impairment. Neuropathol Appl Neurobiol. 2008;34:145‐154. 17973907 10.1111/j.1365-2990.2007.00877.x

[127] Enkhjargal B , McBride DW , Manaenko A , et al. Intranasal administration of vitamin D attenuates blood‐brain barrier disruption through endogenous upregulation of osteopontin and activation of CD44/P‐gp glycosylation signaling after subarachnoid hemorrhage in rats. J Cereb Blood Flow Metab 2017;37:2555‐2566. 27671249 10.1177/0271678X16671147 PMC5531351

[128] Fujimoto M , Takagi Y , Aoki T , et al. Tissue inhibitor of metalloproteinases protect blood‐brain barrier disruption in focal cerebral ischemia. J Cereb Blood Flow Metab. 2008;28:1674‐1685. 18560439 10.1038/jcbfm.2008.59

[129] Petty MA , Lo EH . Junctional complexes of the blood‐brain barrier: permeability changes in neuroinflammation. Prog Neurogibol. 2002;68:311‐323. 10.1016/s0301-0082(02)00128-4 12531232

[130] Yatsushige H , Ostrowski RP , Tsubokawa T , et al. Role of c‐Jun N‐terminal kinase in early brain injury after subarachnoid hemorrhage. J Neurosci Res. 2007;85:1436‐1448. 17410600 10.1002/jnr.21281

[131] Guo Z , Sun X , He Z , et al. Matrix metalloproteinase‐9 potentiates early brain injury after subarachnoid hemorrhage. Neurol Res. 2010;32:715‐720. 19703360 10.1179/016164109X12478302362491

[132] Zuo S , Li W , Li Q , et al. Protective effects of Ephedra sinica extract on blood‐brain barrier integrity and neurological function correlate with complement C3 reduction after subarachnoid hemorrhage in rats. Neurosci Lett. 2015;609:216‐222. 26518242 10.1016/j.neulet.2015.10.056

[133] Mahajan SD , Aalinkeel R , Sykes DE , et al. Tight junction regulation by morphine and HIV‐1 tat modulates blood‐brain barrier permeability. J Clin Immunol. 2008;28:528‐541. 18574677 10.1007/s10875-008-9208-1

[134] Huang B , Krafft PR , Ma Q , et al. Fibroblast growth factors preserve blood‐brain barrier integrity through RhoA inhibition after intracerebral hemorrhage in mice. Neurobiol Dis. 2012;46:204‐214. 22300708 10.1016/j.nbd.2012.01.008 PMC3299916

[135] Ferrara N , Gerber HP , LeCouter J . The biology of VEGF and its receptors. Nat Med. 2003;9:669‐676. 12778165 10.1038/nm0603-669

[136] Kusaka G , Ishikawa M , Nanda A , et al. Signaling pathways for early brain injury after subarachnoid hemorrhage. J Cereb Blood Flow Metab. 2004;24:916‐925. 15362722 10.1097/01.WCB.0000125886.48838.7E

[137] Gavard J , Patel V , Gutkind JS . Angiopoietin‐1 prevents VEGF‐induced endothelial permeability by sequestering Src through mDia. Dev Cell. 2008;14:25‐36. 18194650 10.1016/j.devcel.2007.10.019

[138] Gliem M , Krammes K , Liaw L , et al. Macrophage‐derived osteopontin induces reactive astrocyte polarization and promotes re‐establishment of the blood brain barrier after ischemic stroke. Glia. 2015;63:2198‐2207. 26148976 10.1002/glia.22885

[139] Lee H , Jin Y‐C , Kim S‐W , et al. Proangiogenic functions of an RGD‐SLAY‐containing osteopontin icosamer peptide in HUVECs and in the postischemic brain. Exp Mol Med. 2018;50:e430. 29350679 10.1038/emm.2017.241 PMC5799800

[140] Brenner D , Labreuche J , Touboul P‐J , et al. Cytokine polymorphisms associated with carotid intima‐media thickness in stroke patients. Stroke. 2006;37:1691‐1696. 16741188 10.1161/01.STR.0000226565.76113.6c

[141] van Velthoven CT , Heijnen CJ , van Bel F , Kavelaars A . Osteopontin enhances endogenous repair after neonatal hypoxic‐ischemic brain injury. Stroke. 2011;42:2294‐2301. 21700938 10.1161/STROKEAHA.110.608315

[142] Rabenstein M , Hucklenbroich J , Willuweit A , et al. Osteopontin mediates survival, proliferation and migration of neural stem cells through the chemokine receptor CXCR4. Stem Cell Res Ther. 2015;6:99. 25998490 10.1186/s13287-015-0098-x PMC4464234

[143] Yan YP , Lang BT , Vemuganti R , Dempsey RJ . Persistent migration of neuroblasts from the subventricular zone to the injured striatum mediated by osteopontin following intracerebral hemorrhage. J Neurochem. 2009;109:1624‐1635. 19457159 10.1111/j.1471-4159.2009.06059.x

[144] Yan YP , Lang BT , Vemuganti R , Dempsey RJ . Osteopontin is a mediator of the lateral migration of neuroblasts from the subventricular zone after focal cerebral ischemia. Neurochem Int. 2009;55:826‐832. 19686792 10.1016/j.neuint.2009.08.007

[145] Tao XG , Shi JH , Hao SY , et al. Protective Effects of Calpain Inhibition on Neurovascular Unit Injury through Downregulating Nuclear Factor‐kappaB‐related Inflammation during Traumatic Brain Injury in Mice. Chinese medical journal. 2017;130:187‐198. 28091411 10.4103/0366-6999.198001 PMC5282676

[146] Suzuki H , Shiba M , Fujimoto M , et al. Matricellular protein: a new player in cerebral vasospasm following subarachnoid hemorrhage. Acta neurochir Suppl. 2013;115:213‐218. 22890671 10.1007/978-3-7091-1192-5_39

[147] Suzuki H , Hasegawa Y , Kanamaru K , Zhang JH . Effect of recombinant osteopontin on cerebral vasospasm after subarachnoid hemorrhage in rats. Acta Neurochir Suppl. 2011;110:29‐32. 21125441 10.1007/978-3-7091-0356-2_6

[148] Suzuki H , Hasegawa YU , Chen W , et al. Recombinant osteopontin in cerebral vasospasm after subarachnoid hemorrhage. Ann Neurol. 2010;68:650‐660. 21031580 10.1002/ana.22102 PMC2967465

[149] Mathold K , Wanby P , Brudin L , et al. Alterations in bone turnover markers in patients with noncardio‐embolic ischemic stroke. PLoS One. 2018;13:e0207348. 30496210 10.1371/journal.pone.0207348 PMC6264871

[150] Ozaki T , Muramatsu R , Sasai M , et al. The P2X4 receptor is required for neuroprotection via ischemic preconditioning. Sci Rep. 2016;6:25893. 27173846 10.1038/srep25893 PMC4865734

[151] Li Y , Dammer EB , Zhang‐Brotzge X , et al. Osteopontin is a blood biomarker for microglial activation and brain injury in experimental hypoxic‐ischemic encephalopathy. eNeuro. 2017;4:ENEURO.0253‐16.2016. 10.1523/ENEURO.0253-16.2016 PMC5223053 28101531

[152] Abate MG , Moretto L , Licari I , et al. Osteopontin in the cerebrospinal fluid of patients with severe aneurysmal subarachnoid hemorrhage. Cells. 2019;8:695. 10.3390/cells8070695 PMC6678172 31295895

[153] Carbone F , Busto G , Padroni M , et al. Radiologic cerebral reperfusion at 24 h predicts good clinical outcome. Transl Stroke Res. 2019;10:178‐188. 29949087 10.1007/s12975-018-0637-8

[154] Carbone F , Vuilleumier N , Burger F , et al. Serum osteopontin levels are upregulated and predict disability after an ischaemic stroke. Eur J Clin Invest. 2015;45:579‐586. 25845543 10.1111/eci.12446

[155] Jing M , Li B , Hou X , et al. OPN gene polymorphism and the serum OPN levels confer the susceptibility and prognosis of ischemic stroke in Chinese patients. Cell Physiol Biochem. 2013;32:1798‐1807. 24355932 10.1159/000356613

[156] Kurata M , Okura T , Kumon Y , et al. Plasma thrombin‐cleaved osteopontin elevation after carotid artery stenting in symptomatic ischemic stroke patients. Hypertens Res. 2012;35:207‐212. 22113358 10.1038/hr.2011.177

[157] Kadoglou NPE , Gerasimidis T , Golemati S , et al. The relationship between serum levels of vascular calcification inhibitors and carotid plaque vulnerability. J Vasc Surg. 2008;47:55‐62. 18178454 10.1016/j.jvs.2007.09.058

